# Direct Generation of High-Aspect-Ratio Structures of AISI 316L by Laser-Assisted Powder Deposition

**DOI:** 10.3390/ma13245670

**Published:** 2020-12-11

**Authors:** Piera Alvarez, M. Ángeles Montealegre, Francisco Cordovilla, Ángel García-Beltrán, Ignacio Angulo, José Luis Ocaña

**Affiliations:** 1 Ikergune, Inzu Group, San Antolín 3, 20870 Elgoibar, Spain; 2 Talens Systems, Inzu Group, Polígono Albitxuri 20, 20870 Elgoibar, Spain; mamontealegre@talenssys.com; 3 UPM Laser Centre, Escuela Técnica Superior de Ingenieros Industriales, Polytechnical University of Madrid, C./José Gutiérrez Abascal 2, 28006 Madrid, Spain; francisco.cordovilla.baro@upm.es (F.C.); agarcia@etsii.upm.es (Á.G.-B.); ignacio.angulo@upm.es (I.A.); jlocana@etsii.upm.es (J.L.O.)

**Keywords:** laser metal deposition (LMD), additive manufacturing, mechanical properties, tensile strength, 316L stainless steel, metallic powder, cladding

## Abstract

The effect of process parameters and the orientation of the cladding layer on the mechanical properties of 316L stainless steel components manufactured by laser metal deposition (LMD) was investigated. High aspect-ratio walls were manufactured with layers of a 4.5 mm wide single-cladding track to study the microstructure and mechanical properties along the length and the height of the wall. Samples for the tensile test (according to ASTM E-8M-04) were machined from the wall along both the direction of the layers and the direction perpendicular to them. Cross-sections of the LMD samples were analyzed by optical and scanning electron microscopy (SEM). The orientation of the growing grain was observed. It was associated with the thermal gradient through the building part. A homogeneous microstructure between consecutive layers and some degree of microporosity was observed by SEM. Uniaxial tension tests were performed on samples extracted from the wall in perpendicular and parallel directions. Results for ultimate tensile strength were similar in both cases and with the wrought material. The σ_0.2_ were similar in both cases but slightly superior to the wrought material.

## 1. Introduction

Nowadays, one of the most interesting processes to research in the additive manufacturing (AM) field is the process known as laser metal deposition (LMD) or laser cladding (LC), due to its versatility [1,2] for manufacturing parts with complex structures for industry, minimizing waste material [3,4]. The process is included among the rapid prototyping technologies in its approach to manufacturing a solid component by layer additive. One of the main benefits of LMD is the reduction of material losses in comparison with other approaches such as milling or turning. The difference is that in LMD, the designed geometry is manufactured by adding layers of material, and, on the contrary, in conventional technologies, the material is removed from a solid part. In LMD, a metallic powder is injected through a coaxial nozzle onto a substrate; a high-power laser is used as a heat source to melt the powder together with the substrate generating a metallurgic bond between them, guaranteeing good adhesion between both. The nozzle travels in a relative movement with the substrate, resulting in a single-clad layer after solidification of the melt pool. The overlapping of the single-clads forms a coating, and a 3D structure as the wall studied in this work can be built up layer-by-layer.

Furthermore, LMD has specific characteristics of interest in comparison with another deposition process since it is inherently a low heat input process, resulting in fine microstructures, small heat-affected zones, leading to improve mechanical properties and low distortion [5,6].

The application of LMD in industry has experienced significant development in the last two decades [1,2]. The expansion of LMD involves many areas, such as optimization of process parameters [7,8], simulation [9,10,11]; studies of online control of the LMD process which have in consideration the control of laser powder [12] or the temperature in the melt pool [13], material microstructure [6,14] and mechanical response of the consolidated structure, as well as the behavior of the combination between the deposited material with the substrate, in which the material forms the coating [15,16].

Interesting applications for LMD are to improve the wear or corrosion resistance of surface parts [17] and their use to repair damaged surfaces [18,19]. Tool and die lifetimes can be extended, adding over their surface new layers or repairing their surface with the LMD process [20]. Furthermore, one of the main goals of building pieces by the LMD process is to manufacture solid structures with mechanical properties comparable or even improved with respect to the pieces manufactured following conventional techniques.

There are some studies in which the effect of processing parameters in microstructure and mechanical properties of additive manufacturing stainless steel has been studied [21,22,23]. The literature is mainly focused on studying the mechanical properties of LMD pieces manufactured as a solid part in which overlapped tracks, and the layer-by-layer evolutions have an influence on them [14,24,25,26] even in manufactured pieces with some post-treatment of relieving tensile strength. This work is focused on the study of the mechanical properties of the material after building a wall with a unique laser track as a width.

Temperature monitoring and control in the additive manufacturing technologies has been a topic of interest in the last years: there are some methods for controlling or measuring the melt pool temperature, by applying a single-color infrared pyrometer integrated into a powder feeding nozzle [27], CCD camera to measure the melt pool [28] used to control the height of the cladding.

The focus on high aspect ratio walls implies specific challenges due to several factors such as the control of high powder flows, the heat accumulation in the sample during the process and the homogeneity of the microstructure along the profile of the wall.

This paper describes our research to understand the mechanical behavior of LMD-like walls manufactured with only one laser clad as width and without any post-treatment. The hardness and tensile strength in the generated structure have been studied in two directions of the manufactured wall; parallel and perpendicular to the deposited layers. The tensile strength of the perpendicular studies has resulted in better behavior in comparison with the parallel orientation.

The formation of porosity is a typical defect that can appear in LMD manufactured pieces. Due to the bad effect that the porosity has on the mechanical properties [29]. The study of porosity has been a point of discussion in some papers, focusing on studying the causes of porosity and developing models to predict it [15,23].

The microstructure observed along the cross-section of the wall reveals a homogeneity and continuity among the deposited layers, although some microporosity was observed by scanning electron microscopy.

This paper aims at advancing the knowledge of the properties of the additive manufactured material as a direct result from the process, in contrast with applying any kind of post-processing, which is associated with poor productivity and high costs.

## 2. Materials and Methods

### 2.1. Additive Powder

**Figure 1:**
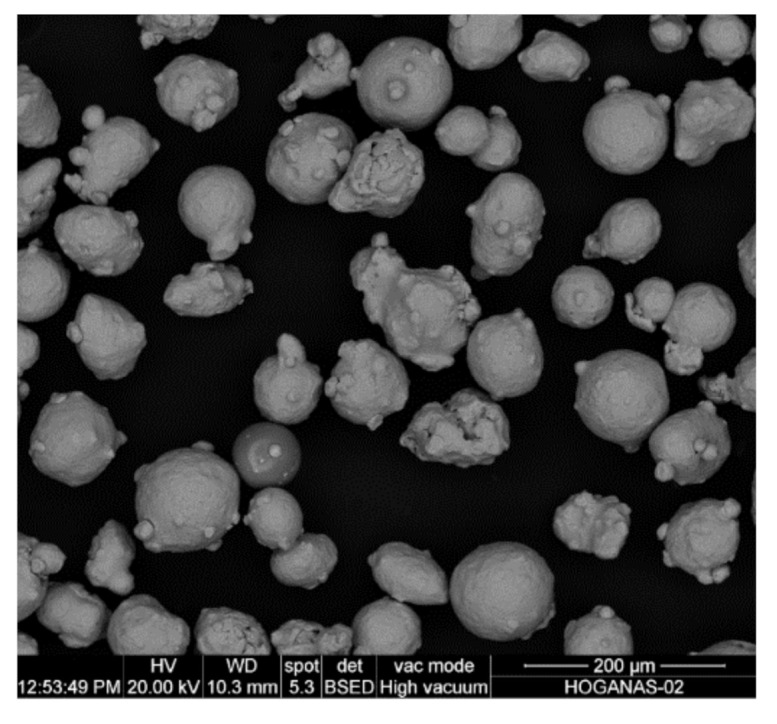
SEM micrograph of the AISI 316L stainless steel powder.

The powder used in this analysis was gas atomized AISI 316L, supplied by Höganäs, with a particle size value between 44 and 106 µm. Figure 1 shows an SEM micrograph of the powder, and the corresponding chemical composition is listed in Table 1.

### 2.2. Experimental Procedure

LMD experiments were carried out by using a high-power fiber laser IPG YLS-6000 (λ = 1070 nm), with a 5 mm diameter, as can be seen in [7].

####  The Optical Head was Mounted in a 3 Linear Axis System

**Figure 2:**
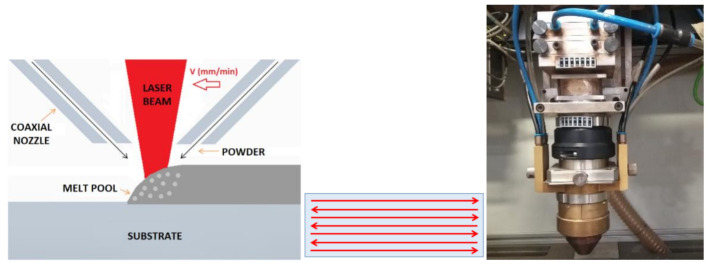
Scheme of the coaxial cladding system. Laser metal deposition (LMD) strategy for building the wall and powder nozzle used in the experiment [7].

Metallic powder particles were placed in a GTV powder feeder and delivered to the process zone through a coaxial nozzle, Powerline12 from Fraunhofer IWS, using nitrogen as a carrier and shielding gas. A scheme of the coaxial cladding system is in Figure 2. A zigzag-like moving strategy was followed to build a single wall. In Figure 2 center, the arrows show the direction of the laser along each layer.

The impact of the laser parameters such as travel speed, laser power, or powder flow on the geometrical dimensions of the single-clad deposited is, generally, well known [30,31]. As a general tendency, low powder flows and process speeds melt the substrate material, thus increasing the degree to which a given layer penetrates into the previous one or into the substrate, known as dilution [32].

**Figure 3:**
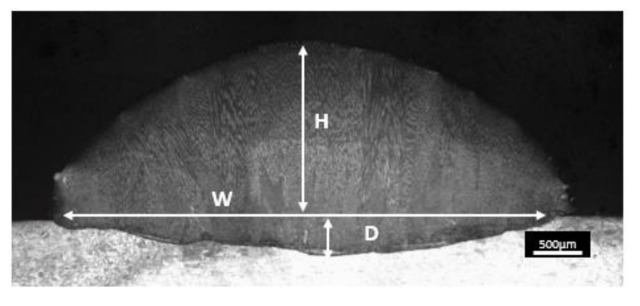
Optical microscopy (OM) of a single-clad showing the geometrical measurements.

Before manufacturing the solid part by LMD, in the first place, single-clads were characterized, in which laser power and process speed have varied [7]. Then, the final measurements of the manufactured single-clads (dilution, width and height) were determined in a cross-section of the clads (Figure 3). These combinations of process parameters and dimensions of the single-clads were taken as a reference to characterize the layers with which the walls were built. During the laser process, a thermographic camera Optris PI400 calibrated until 1800 °C, was used for monitoring the temperature of the process at each moment.

Conventional metallography techniques (grinding and polishing) were used to prepare the samples for microstructural characterization. After mirror polishing, the surface was etched with V2A acid, a special reagent for stainless steel, to reveal the microstructure.

Cross-sections of the single-clads were prepared to be observed by optical microscopy (OM) using an Optika brand device, which has an in-built camera Leica DMC2900. For more detail, the fracture area of the tested samples was observed by a scanning electron microscopy (SEM) Hitachi s300n model.

A range of laser power between 2600 and 3000 W and process speed between 10 and 17 mm/s were used in the present study giving values of energy density between 30 and 50 J/mm^2^. For this work, the powder flow (25 g/min), shielding gas (8 L/min) and carrier gas (4 L/min) values were kept constant.

**Figure 4:**
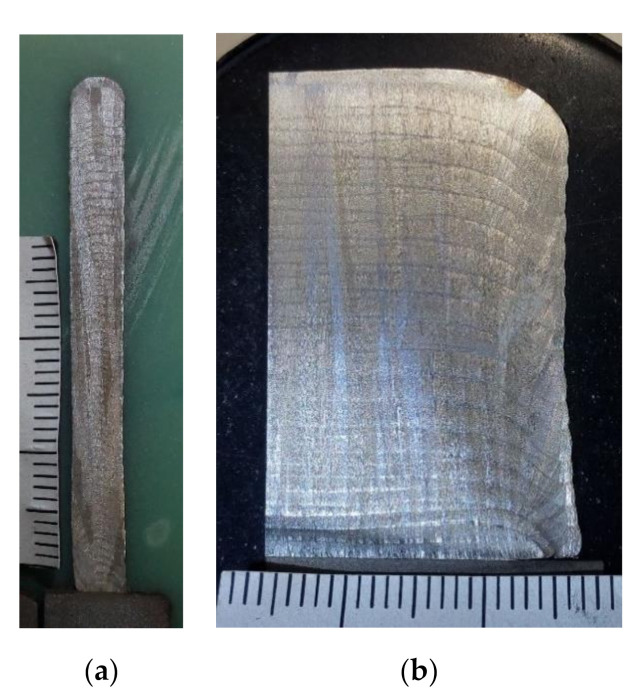
Cross-section of the additive material perpendicular (**a**) and parallel to the layers (**b**).

Previous to machining the tensile-test samples from the fabricated wall, the powder deposited layer-by-layer, using the range of optimized parameters, in order to build solid structures to analyze their homogeneity, geometrical properties, continuity between layers, porosity, etc. As an example of the pieces studied, cross and transversal sections of the manufactured LMD walls are shown in Figure 4. Although the deposited layers can be distinguished, in general, homogeneity is observed between them, an absence of large pores or zones with unmelted particles, which are the cause of lack of fusion between layers that can decrease the mechanical behavior.

Using a microhardness tester FM-300, microhardness in Vickers units was measured along the cross-section by applying a 300 g load and a dwell time of 12 s.

**Figure 5:**
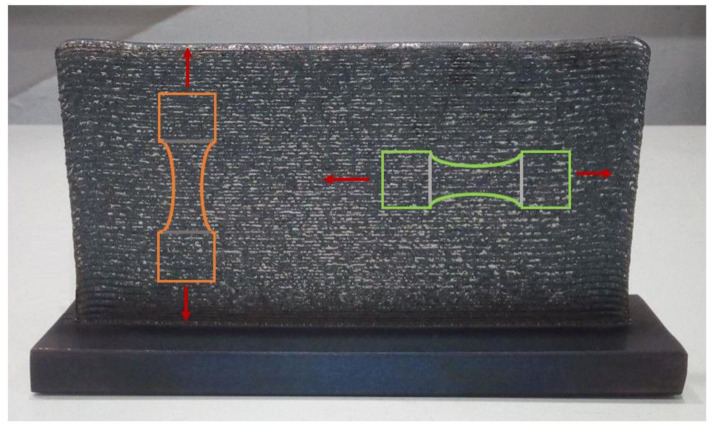
LMD wall manufactured. Orange and green figures denote the direction of the specimens, perpendicular and parallel, respectively. Red arrow shows the direction of strength.

Some walls were built with a width of only one cladding track (4.5 mm) layer-by-layer until the necessary dimensions were achieved to machine the tensile test specimens. The dimensions of the manufactured wall were 4.5 × 180 × 110 mm^3^, Figure 5.

To build a high wall like the one studied in this study, the working distance is also a parameter to take into account to avoid the defocusing of the laser spot, which could generate an oversizing in the melting pool. It is necessary to maintain it to control the width of the melting pool and, therefore, the width and height of the wall.

In the case of an over-focus condition, the focal plane is located above the substrate, resulting in an excessive defocus, which can permit the laser beam to shine down the edges of the component, increasing the melting pool that allows the material to fall. If this occurs, the layer could have heterogeneous height, which has an influence on the good building of the wall.

As mentioned above, laser power and processing speed are the most important parameters to consider for construction accuracy. They have an influence on the heat input and the heat dissipation and further influence the evolution of microstructure and macrostructure shape precision of the part in the LMD process [33].

Both parameters were adapted with the growth of the layer in order to control the temperature of the process and, in consequence, to control the microstructure and to maintain constant the width of the melting pool along the height of the wall. A big increase in temperature has a negative effect on the accuracy of the geometry of the wall, causing the fall of the wall or an increase in width due to overheating. The dynamic viscosity of the liquid metal increases, notably when its temperature exceeds the melting point of the material.

Figure 5 shows the LMD wall manufactured for this study. A homogeneous structure and height can be seen along the whole wall. Orange and green schemes inside show the orientations in which the tensile test specimens were machined. Red arrows show the direction of the uniaxial tensile tests.

**Figure 6:**
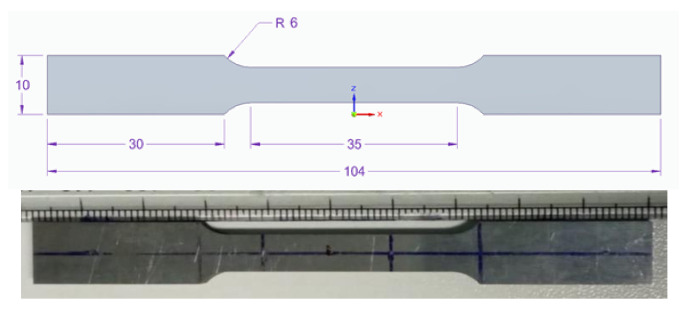
Scheme and tensile specimen studied. The thickness of the specimens was 4 mm.

To improve the surface quality of the additive manufactured steel, a dry milling finish was applied on both surfaces of the piece. Tensile test specimens were designed according to the ASTM E-8M-04 standard. The specimens were sectioned from the additive manufactured stainless steel samples by wire electrical discharge machining (WEDM). As explained in the experimental part, the building direction is the same for all samples. The difference between the specimens studied is the direction in which the tensile samples were extracted. Orange samples are denoted perpendicular (the applied strength is perpendicular to the layers) and green sample parallel (the applied strength is parallel to the layer). Figure 5 shows the homogeneous additive part from which the specimens were machined, five specimens for each condition were tested. A scheme with the dimensions of the samples and a machined sample are shown in Figure 6.

Tensile tests were made using a 100 kN 810 MTS system. An extensometer was used to record the deformation of samples with a gauge length of 20 mm (MTS model 634.31 F-24).

## 3. Results and Discussion

### 3.1. Microstructure Characterization

**Figure 7:**
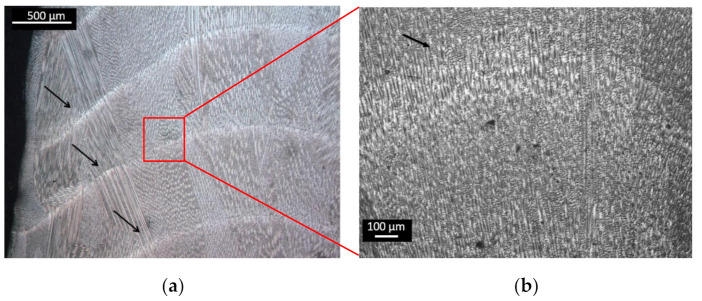

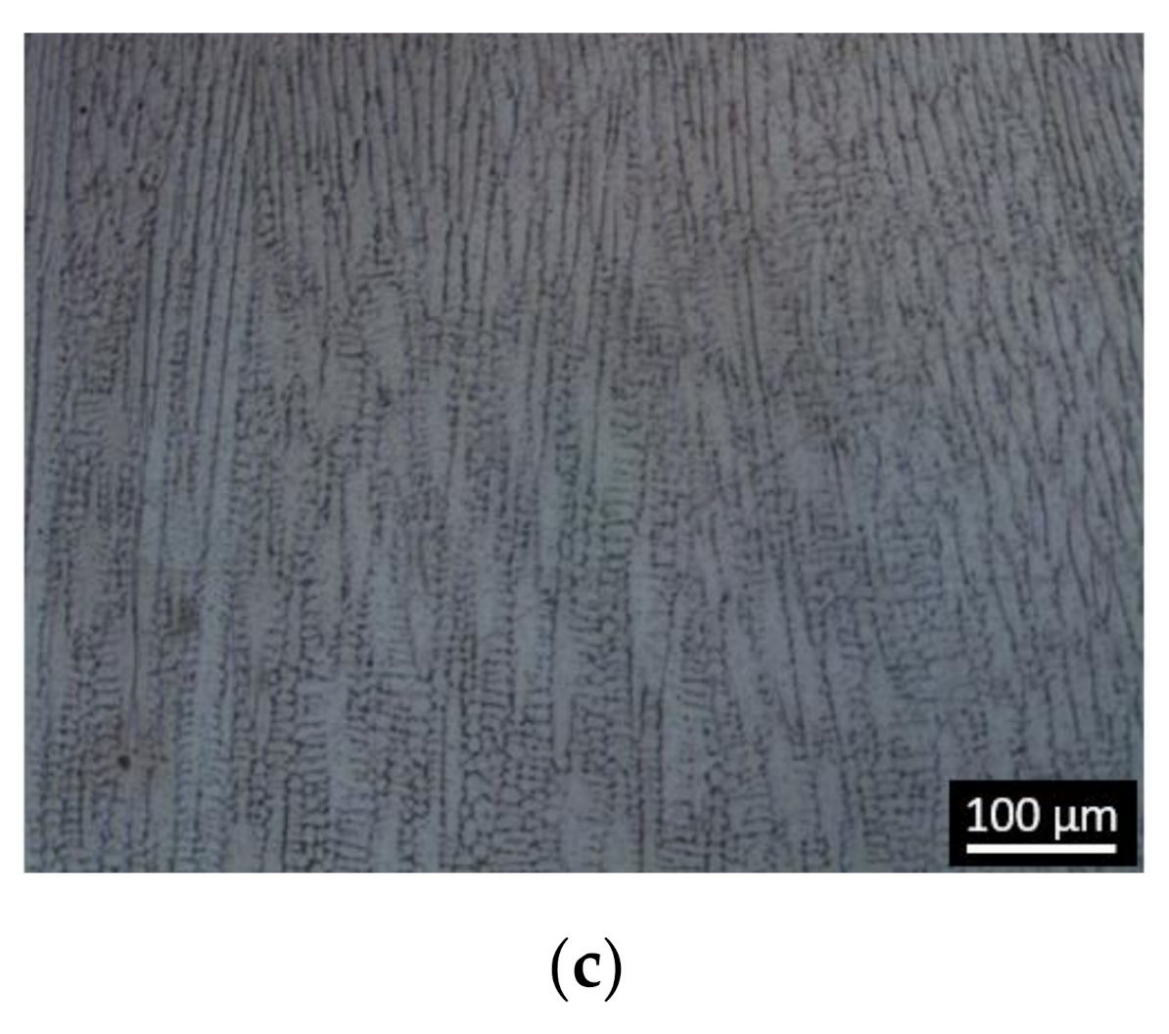
Optical micrographs of a detail of the cross-section of the wall (perpendicular to the layers). (**a**) The arrows show the limit between different layers. (**b**) Detail of the microstructure between layers. (**c**) Grain oriented along the predominant heat flow from the bottom to top in the micrograph.

A homogeneous microstructure was observed along the cross-section of the LMD wall manufactured. Directional growth of the grain could be observed through the border of the wall due to the thermal gradient where the dissipation of heat occurs. The bonding between consecutive layers was observed, with a continuity of the grains, Figure 7a. Continuity of the microstructure along the different layers was observed in Figure 7b. In more detail, the oriented grains were seen in Figure 7c, where the direction of the heat flow was from bottom to top in the micrograph. Dendrites morphology was observed, forming columnar grains oriented along the building direction due to the solidification direction during the LMD process. In austenitic stainless steels deposited by LMD, microstructural grains and ferrite dendrites were preferentially oriented along the highest thermal gradient [34]. The orientation of the grains and dendrites had an influence on the properties of the additive piece, so this orientation of the grains had an influence on the mechanical properties as well as regarding the orientation of the tested pieces, parallel or perpendicular to the layers.

**Figure 8:**
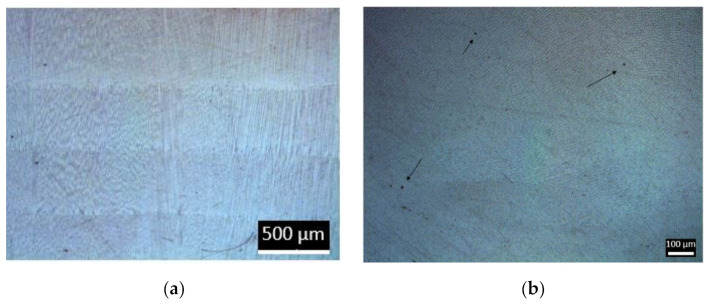
Optical micrographs of the wall: (**a**) parallel to the deposited layer; (**b**) perpendicular to the deposited layer.

Figure 8 shows two optical micrographs of the wall in different orientations, (a) parallel layers and (b) perpendicular to the layers. In the parallel section, the limit of the layers can be observed with a homogenous microstructure. In the perpendicular layer, a cellular microstructure was observed, resulting in the cutting of the grains, which were oriented in the direction of growing the wall. Small pores can also be observed in Figure 8b.

**Figure 9:**
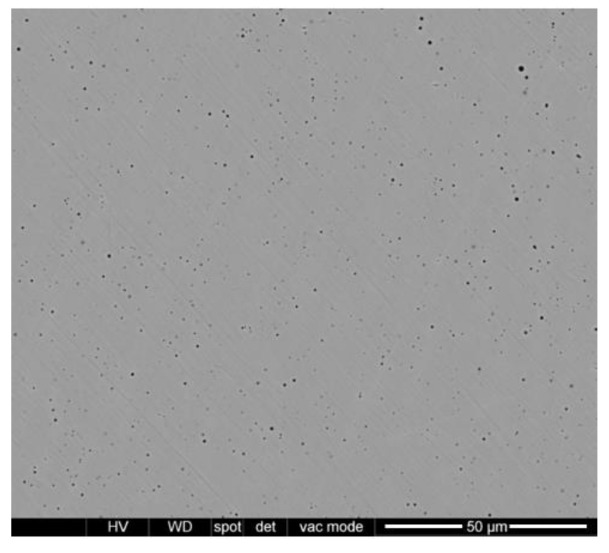
SEM, microporosity observed in cross-section.

Some microporosity was observed in the cross-section of the LMD structure (Figure 8). The morphology of gas pores was typically spherical, and gas porosity appears anywhere in a component, as observed in Figure 9. Similar to what occurs in laser welding, the gas trapped during the process had no time to evacuate before the next layer, so it was entrapped in the melt pool due to the rapid solidification during the LMD process. It was important to note that the diameter of the pores was less than 5 μm. To quantify the porosity, samples were weighed after manufacturing in the final machining of them. Their volume was known from the CAD, which, additionally, was checked with accurate measurements of the real specimens. From those results, the real density of the sample was determined as 97% of the density of the bulk material. This slight difference between the bulk material and the additive manufactured parts could be related to the small size of the pores. Although there was a noticeable amount of pores, as shown in Figure 9, their small diameter, around 1–2 µm, had a limited impact on the global density of the sample.

### 3.2. Mechanical Properties

The study of the mechanical response of the consolidated structures is one of the main points to have information about the quality of the LMD process. Hardness (according to the Vickers scale) and tensile strength experiments are performed to determine key properties on components fabricated by additive manufactured technologies.

**Figure 10:**
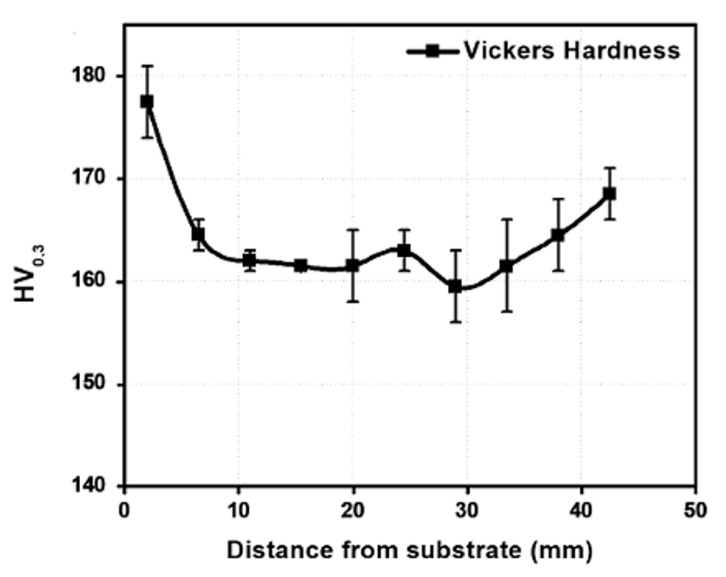
Hardness values in a cross-section of the wall from the bottom to the top.

Hardness values were carried out in the transversal section of the LMD wall from the area near the substrate to the top of the wall. Figure 10 shows the curve of hardness values, an average of three values taken in different areas of the piece built. Moreover, along the structure, some discontinuities in the microstructure could be found. The indentations to measure the hardness were made randomly along the surface, so if the indentation is in the middle of a layer or in the intersection between two layers, there could be a variation of the hardness values. For this reason, the uncertainty values are also shown in the graph by the straight line. As can be seen in Figure 10, the values were quite similar along the wall, which means that the additive material had a homogeneous microstructure along the manufactured piece. However, the highest values were found near the substrate. This was due to the fact that the cooling speed around the substrate was higher due to the dissipation of heat by conduction through the substrate resulting in a finer microstructure in this area. An increase in the hardness was also observed at the top of the structure due to the higher cooling rate in this area. In any case, the tensile test specimens were machined from the center of the wall, where the hardness values were homogeneous.

**Figure 11:**
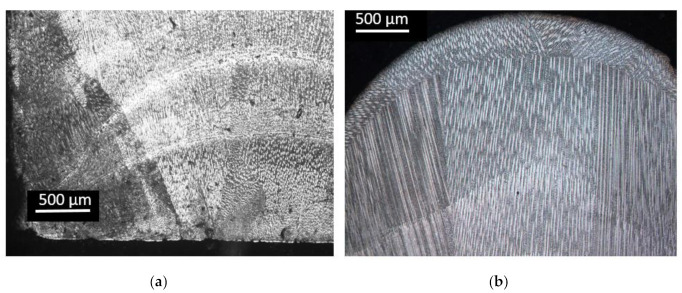
Optical micrographs of the cross-section of the wall (**a**) at the bottom, close to the substrate, and (**b**) on the top of the wall.

Figure 11 shows the microstructure observed in a cross-section of the manufactured wall, at the bottom of the wall, close to the substrate where a certain diffusion of carbon can occur in the deposited layer, causing, together with the finer microstructure, the increase in hardness value observed in this zone.

#### Tensile Tests

**Figure 12:**
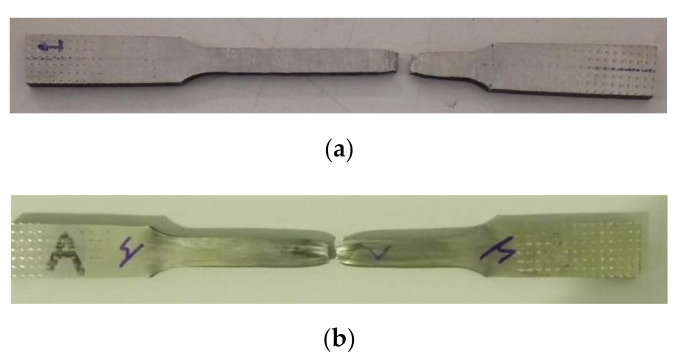
Photograph of two tensile test specimens after being tested. (**a**) In perpendicular and (**b**) parallel directions.

Two photographs of tensile test specimens after testing are shown in Figure 12. In both cases, perpendicular and parallel, a small reduction of the cross-section was achieved, as typically observed in this kind of test in the broken area, and both samples had broken close to the middle of the calibrated length. The poor contraction in the zone of the fracture was also observed.

**Figure 13:**
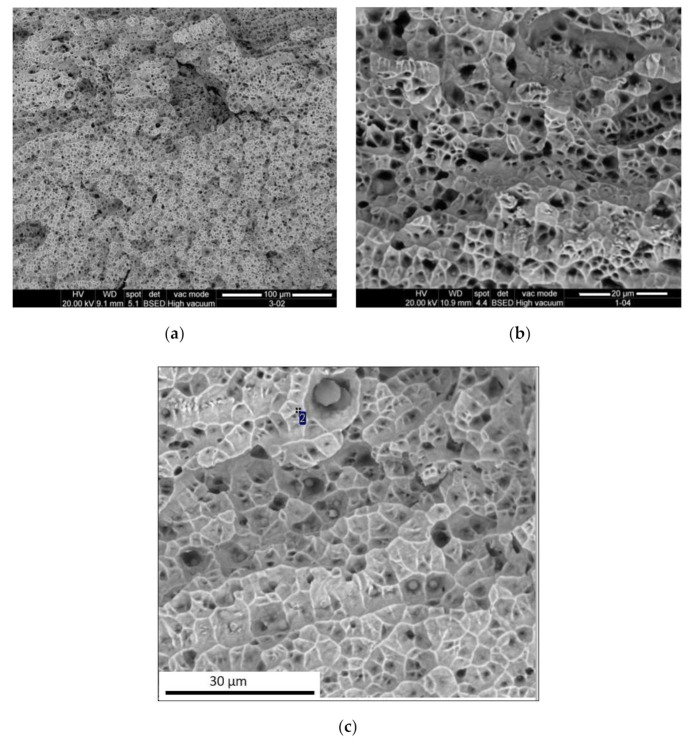
(**a**–**c**) SEM after tensile testing.

Figure 13 shows the SEM fractographies of the samples after tensile testing. Dimple morphology dominates on the fracture surface of all samples, which is indicative of a ductile fracture mode. Small particles were found inside the dimple of the fracture (Figure 13c), similar to that observed by other authors [35], which was related to oxides. These internal particles can affect the mechanical properties and be the cause of the minor values of yield strength obtained in this study.

**Figure 14:**
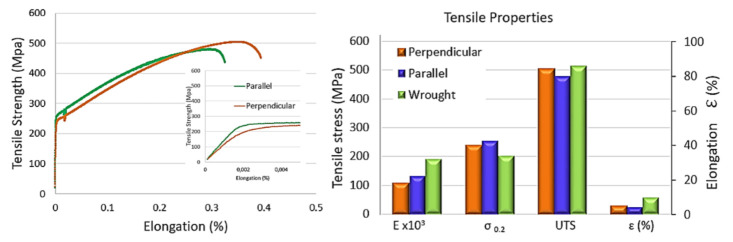
Mechanical properties of LMD samples manufactured at different orientations of the layer compared with the material in wrought condition [37].

To study the mechanical behavior of the wall generated layer-by-layer with only one laser track, uniaxial tension tests were performed to determine the ultimate tensile strength (UTS), yield strength (σ0.2) and elongation at break (ε). LMD-like standard tensile test specimens extracted at different orientations: parallel and perpendicular to the layers were studied, meaning, with any post-treatment. It should be noted that the data were obtained by mathematically averaging the test results of five samples for each direction extracted from the same wall. Figure 14 shows the curve and results of tensile properties. LMD samples have reduced tensile test and relative elongation as compared to the wrought stainless steel. The values are similar to those found in additive manufactured samples at a high process speed [14]. With respect to the orientations studied, samples extracted in perpendicular orientation have higher UTS than the sample extracted in a parallel orientation, while less yield strength is observed in perpendicular orientations similar to those obtained in [36] (Yield strength 207–261 and UTS 414–539), also for perpendicular orientation. Similar elongation is observed in both orientations, being higher for the perpendicular orientation, 30%, than parallel orientation, 25% of relative elongation.

Table 2 summarizes the tensile properties obtained for the five specimens tested in different directions, parallel and perpendicular to the deposited layer. The average values and the standard deviation were also calculated. As it can be observed, the value of standard deviation was comparably low. These results confirm the homogeneity of the microstructure along the manufactured wall in both directions of the deposited layers.

### 3.3. Discussion

The objective of this work was to study and analyze the mechanical behavior of the material in an LMD-like condition, i.e., without applying any post-treatment of the manufacturing, which allowed the mechanical response of the material to be shown as it was directly obtained. The advantage of this study was to have information about the material and its performance without any kind of post-processing.

**Figure 15:**
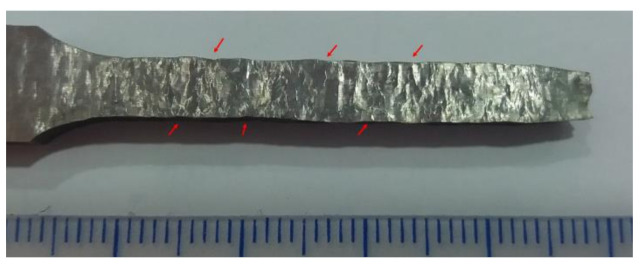
Surface of the calibrated longitude of the tensile specimen after being tested. Red arrows show the striations.

According to the standard specification of ASTM (UTS 480 MPa and σ_0.2_ 205 MPa) for wrought 316L stainless steel [38], UTS and σ_0.2_ achieved in both studied conditions (perpendicular and parallel) are higher. The Young’s modulus of the specimens studied in this work was slightly lower, as was the relative elongation. These lower properties were attributed to the emergence of some striations, which could be observed in the tested zone of the perpendicular samples. This phenomenon was associated with the deformation of the layers deposited during the tensile test. At the side of the tested sample, some oscillations were noticed, with alternate narrowing of the sample along the stretched length, as can be observed in Figure 15, indicated by red arrows.

The values achieved in the specimens manufactured in the present study were lower in comparison with the results in [18], where the mechanical properties of the cladding of AISI 316L in different orientations were studied. The reason for these values can be found in the strategy followed to build the additive piece from which the samples were machined. In the current paper, a wall of a single track was built, while in the previously mentioned paper, an AM structure was obtained, so there was more influence on the different layers and with the overlapping of the consecutive claddings.

Additionally, the smaller values found in this study at the yield stress with the perpendicular samples were interpreted as a result of the accommodation of the consecutive layers during the tensile test. This could be due to the microporosity found in the additive material, observed by SEM (Figure 13). The value of yield strength, less than conventional AISI 316L, was attributed to this presence of porosity. The tested section was shorter than the effective section of the sample.

## 4. Conclusions

The aim of the present paper was to examine the microstructural and mechanical behavior of an LMD structure built layer-by-layer with a single laser track of 4.5 mm width. Microstructure, hardness and tensile strength were studied in different orientations of the cladding. The results obtained for UTS were similar in both studied cases depending on the direction of the deposited layers, being 506 MPa for perpendicular and 491 MPa for parallel direction. Comparing the yield strength for parallel directions was found to be slightly higher, 253 MPa, while for perpendicular direction, it was 239 MPa. Comparing these values with the values in the bibliography for wrought material, it can be concluded that the manufactured-like wall has similar mechanical properties. Even the yield strength was slightly higher than the wrought material.

Hence, in this study, walls were built with mechanical properties comparable to the wrought material; in spite of the microporosity observed in scanning electron micrographics, the UTS in both orientations and wrought material are in the same order of magnitude.

## Figures and Tables

**Table 1:** Chemical composition (wt.%) of the 316L stainless steel.

Material	C	Cr	Ni	Mn	Mo	Si	Fe
AISI 316L	0.02	17	12	1.5	2.5	0.8	Balance

**Table 2:** Tensile tests on LMD specimens.

#Samples	E (GPa)		σ_0.2_ (MPa)		UTS (MPa)		ɛ (%)	
	Perpendicular	Parallel	Perpendicular	Parallel	Perpendicular	Parallel	Perpendicular	Parallel
1	105	110	238	250	505	450	28	25
2	97	125	230	268	479	503	32	25
3	93	122	244	255	524	490	30	26
4	91	107	243	242	524	482	32	26
5	95	112	237	249	498	481	30	25
Average	96	115	239	253	506	491	30.4	25.3
Standard Deviation	5.5	7.7	5.6	9.5	19	19.5	1.7	0.5
